# Opening up connectivity between documents, structures and bioactivity

**DOI:** 10.3762/bjoc.16.54

**Published:** 2020-04-02

**Authors:** Christopher Southan

**Affiliations:** 1 Deanery of Biomedical Sciences, University of Edinburgh, Edinburgh, EH8 9XD, UK; 2 TW2Informatics Ltd, Västra Frölunda, Gothenburg, 42166, Sweden

**Keywords:** activity data, databases, drug discovery, chemical structures, protein targets

## Abstract

Bioscientists reading papers or patents strive to discern the key relationships reported within a document “D“ where a bioactivity “A” with a quantitative result “R” (e.g., an IC_50_) is reported for chemical structure “C” that modulates (e.g., inhibits) a protein target “P”. A useful shorthand for this connectivity thus becomes DARCP. The problem at the core of this article is that the community has spent millions effectively burying these relationships in PDFs over many decades but must now spend millions more trying to get them back out. The key imperative for this is to increase the flow into structured open databases. The positive impacts will include expanded data mining opportunities for drug discovery and chemical biology. Over the last decade commercial sources have manually extracted DARCP from ≈300,000 documents encompassing ≈7 million compounds interacting with ≈10,000 targets. Over a similar time, the Guide to Pharmacology, BindingDB and ChEMBL have carried out analogues DARCP extractions. Although their expert-curated numbers are lower (i.e., ≈2 million compounds against ≈3700 human proteins), these open sources have the great advantage of being merged within PubChem. Parallel efforts have focused on the extraction of document-to-compound (D-C-only) connectivity. In the absence of molecular mechanism of action (mmoa) annotation, this is of less value but can be automatically extracted. This has been significantly accomplished for patents, (e.g., by IBM, SureChEMBL and WIPO) for over 30 million compounds in PubChem. These have recently been joined by 1.4 million D-C submissions from three major chemistry publishers. In addition, both the European and US PubMed Central portals now add chemistry look-ups from abstracts and full-text papers. However, the fully automated extraction of DARCLP has not yet been achieved. This stands in contrast to the ability of biocurators to discern these relationships in minutes. Unfortunately, no journals have yet instigated a flow of author-specified DARCP directly into open databases. Progress may come from trends such as open science, open access (OA), findable, accessible, interoperable and reusable (FAIR), resource description framework (RDF) and WikiData. However, we will need to await the technical applicability in respect to DARCP capture to see if this opens up connectivity.

## Introduction

This article assesses a key aspect of data sharing that has the potential to accelerate the progress and impact of medicinal chemistry. To achieve this the community needs to increase the outward flow of experimental results locked-up in millions of published PDFs into structured open databases that explicitly capture the connectivity between structures, documents and bioactivity results. But isn’t there enough of this out there already? This can be answered in two parts. The first is that a conservative estimate of the capture backlog would be at least two-fold more data still entombed in PDFs that is not currently indexed in database records. The second part is the imperative to enable open science data mining at all scales. This applies not only to individual documents (i.e., small data) but scaling up to all papers and patents (i.e., big data). The potential of the latter is huge, especially since artificial intelligence (AI) is being increasingly applied to knowledge distillation. This report will outline the principles of connectivity capture, selected sources, progress, impediments and prospects for their amelioration.

## Review

### Defining terms

It is necessary to outline the topics covered:

**Medicinal chemistry:** As directed towards drug discovery this needs no introduction. However, in the broader context of bioactive chemistry, it becomes indivisible from the related domains of chemical biology (directed towards mechanistic insight rather that direct drug discovery), enzymology, pharmacology, and toxicology in addition to the development of insecticides or herbicides.

**Connectivity:** This term is used for an explicit link (e.g., a URL) between a published document and the chemical structures specified therein. Implicit is not only manual navigation (e.g., link-clicking) but also that such connectivity can be made machine-readable and thus computationally interrogated at large scale via an application programming interface (API) or a resource description framework (RDF).

**Papers as documents:** This typically refers to research papers from journals but increasingly needs to encompass their associated supplementary data. Note also that by far the majority of medicinal chemistry, biological chemistry and pharmacology papers are still behind subscription paywalls. However, the full-text for some of them is not only open but also available to be mined in both PubMed Central (PMC) [1] and European PubMed Central (EPMC) [2]. Connectivity can extend to other document types such as review articles and vendor catalogues. In this article the main document type referred to will be the PubMed identifier (PMID). These have open abstracts and are also indexed in the digital object identifier system (DOI). However, significant numbers of papers in the bioactive chemistry domain (including preprints) may be DOI-only.

**Patents as documents:** Academics tend to overlook that patents a) include several fold more medicinal chemistry than papers b) appear years earlier c) most academic drug discovery operations apply for them d) they include a proportion of high quality data that never appears in journals e) they can be text-mined and d) consequently, over 30 million structures have entered PubChem via automated extraction [3].

**Non-document sources:** While this article has to be restricted to documents an increasing amount of drug discovery data is beginning to surface on the web that may never be instantiated in document form. Although this started with PubChem Bioassay as far back as 2004, the more recent proliferation is via open-notebook science. More projects are using open electronic laboratory notebooks (ELNs) that are not only accessible to anyone by web browsing, but also, crucially, crawled by Google and indexed for chemistry searching [4–5].

**Structures:** A necessary focus of this article will be traditional small-molecule chemistry that is not too far outside the rule-of-five lead-like property space. In terms of connectivity antibodies, other protein biotherapeutics, as well as large peptides or polynucleotides, are also important to encompass. However, capture into structured records is more challenging for these larger therapeutic modalities than for small-molecules that can be merged on the basis of chemistry rules. Notwithstanding, space limitations mean that non-small molecule connectivity is out of scope for this article.

**Bioactivity:** This covers a wide spectrum of assay read-outs but with a focus on in vitro, in cellulo, in vivo and in clinico. Ideally this should also include low or inactive analogues which are crucial for SAR elucidation but documents are (understandably) biased towards positive results.

**Open:** As the theme of this special issue this term will doubtless be expanded on in other articles. However, brief qualification in the context of this work is necessary. Regardless of licensing complications, open is taken here to mean public data sources accessible via a web browser (signing in may be an impediment but not a stopper). These are thus distinct from commercial offerings where access has to be purchased.

### Relationship representation

**Figure 1:**
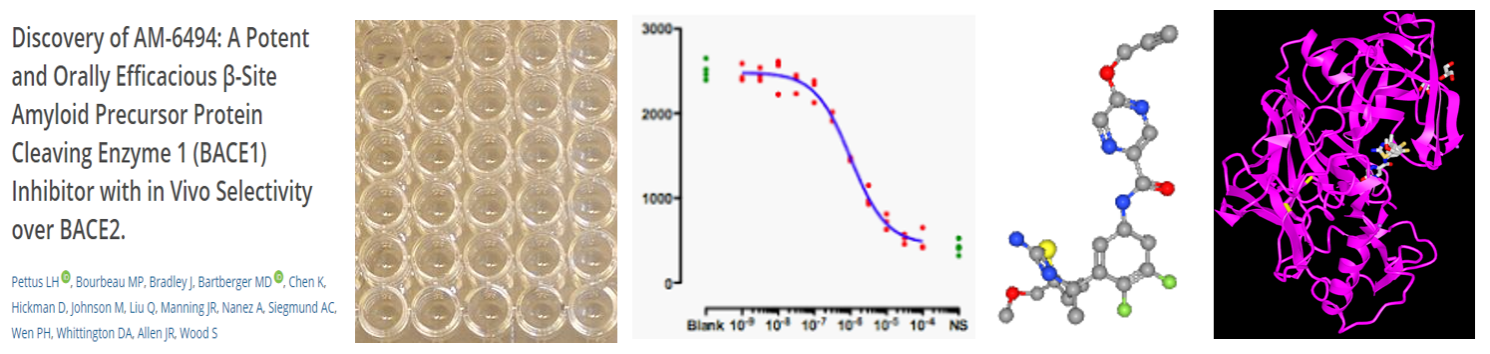
A schematic of the document > assay > result > compound > protein target relationships, D-A-R-C-P [microplate photo adapted from https://commons.wikimedia.org/wiki/File:Microtiter_plate.JPG by Jeffrey M. Vinocur, source licensed under the Creative Commons Attribution Generic 2.5 license (CC-BY 2.5), https://creativecommons.org/licenses/by/2.5/].

As outlined in the abstract the connectivity between documents, structures and bioactivity can be expressed in shorthand as “D-A-R-C-P” (DARCP). This is shown schematically in Figure 1.

The entity specifications can be adapted to different use-cases. For example, the substitution of “P” with target “T” can be used where “T” is a cell or a microorganism. Another example would be where an SAR series can be represented as a multiplexed set of one-A-to-many-R-C. It can also be extended to “D-A-R-C-L-P” where L refers to the explicit location references for C in the document (e.g., “compound **10b**” in a paper or “example 503” in a patent). However, as a formalism for bioactivity there are exceptions and mechanistic nuances that do not fit a DARCP simplification. An example would be heparin (GtoPdb ligand 4214). This could be a commercial partially purified extract of 1200–1500 Mw which consequently does not have a defined chemical structure as “C”. However, as a curatorial expedient, the chemically defined form (as PubChem CID 22833565 with 1040 Mw) has been annotated (even though the sodium salt is the active form in vivo*)*. Note also that while formally “P” in the heparin case is SERPINC1 (ATIII) the mechanism is an indirect one involving the activation of binding to F2 (activated thrombin) for inhibition. Another problematic example is mechanism-based covalent inhibition where the time dependence of IC50 for “A” is not captured.

The structured capture of DARCLP by curation (or at least DARCP) has very high value from the additional relationships that can be explored via the entities and attributes as outlined below:

- Documents: clustering by content relatedness, position within citation networks, connections via authors or institutional affiliations.
- Assays: classified by various assay ontologies.
- Results: log transformations (e.g., pIC_50_ or p*K*_i_) for potency ranking and implicit molecular mechanism of action (mmoa), (e.g., where A-R indicates C to be a potent inhibitor of P).
- Compounds: a full range of cheminformatic analysis including 2D or 3D clustering, property prediction and chemical ontology assignments.
- Proteins: a full range of bioinformatic analysis including gene ontology (GO) assignments, pathway annotation, structural homology, disease associations and genetic variation (e.g., for target validation).

Those cases where the link is only compound-to-document can be referred to as D-C (or c2d). These have become available in a large excess over full DARCLP since they are technically easier to obtain and can be automated to a usable level of specificity. This needs the introduction of the intuitive concept of “aboutness” (ABNS). The title of a document from which D-C could be extracted usually includes an explicit ABNS statement. For example, the code name of a lead compound would be what a medicinal chemistry journal article would be “about”. In the same way, the ABNS of a clinical pharmacology journal article some years later could be describing the clinical trial results for the identical structure which, by then, could have an international non-proprietary name (INN). However, the extraction of multiple compounds (i.e., one-D-to-many-Cs) immediately becomes problematic in the absence of full relationship chains. For example, the medicinal chemistry article may describe the testing of a useful set of analogues for SAR but (as is usually the case) the A-R-P data was not extracted.

At this point we need to introduce the additional concept of name-to-structure (n2s). This is an important determinant of both ABNS and D-C utility. Using the example again from a paper this would mean that both the code name and the INN would be included in the D-C capture record (i.e., n2s) even if 50 analogues were also tested. Other examples that present particular ABNS problems are review articles, synthetic chemistry papers and patents. A review could exemplify 20 lead compounds all with different company code numbers and/or INNs, an extended synthesis report could give rise to 200 D-C records and a patent could have over 500. Discerning the ABNS for patents can be especially problematic since frankly obfuscatory titles and abstracts are common (e.g., “Novel Compounds”).

### The “hamburger” problem

This can be summarised by the following (unattributed) quote “We have spent millions putting data into the literature but now have to spend millions more getting it back out”. This alludes to entombing the DARCP “meat” within a PDF “hamburger”. The paradox is that electronic text formats typically used for drafting papers are machine-readable (certainly with modern parsing techniques). However, this is systematically obviated by the PDF conversion. For example, a chemist may have SMILEs and/or InChIs in their ELN and/or molfiles in an institutional data repository. However, they have to convert this to a ChemDraw proprietary file format in order to render the structural image that eventually appears in the PDF. This means getting the structures “back out” for database capture needs either manual re-sketching or use of an image-to-structure (i2s) tool such as optical structure recognition (OSRA) [6], both of which are error-prone processes.

The common practice of including tables of Markush representations, while they improve SAR readability, makes the extraction problem worse. While most medicinal chemistry journals will include IUPAC names in the synthesis descriptions, these also have to be pulled “back out” of the PDF. This can be done via PDF-to-text optical character recognition (OCR) or curated by pasting across to the Open Parser for Systematic IUPAC Nomenclature (OPSIN) tool [7]. Here again, both the automatic and manual procedures are error prone. Locally-stored SAR data from an Excel sheet or an ELN can be used to populate draft manuscripts (and with lower error rates) but the irony is conversion to PDF (i.e., entombment) makes the ARC in result tables more difficult to extract.

A specific example of the problem can be given for a 2017 article on new antimalarial compounds entering development [8]. Because the chemistry representations were restricted only to images in the PDF a blog post was necessary to manually map the structures to PubChem identifiers [9]. The MyNCBI link to the 16 CID entries given at the top of the blog post is still live (indicating reassuring persistence for this system after four years). While this initial connectivity was only D-C (and where D was a review article rather than a primary activity report) this example had an important sequel. During the curation of the new Guide to Malaria Pharmacology 14 of the 16 compounds now have full DARCLP annotation where D is the primary activity report, P is the *Plasmodium* target and activity values against the parasite are included in the records [10].

### Commercial capture

Since this report is about open connectivity it might not seem pertinent to review commercial resources. However, a brief assessment of these is relevant in several contexts. The first is that, despite occasional use of the adjective “proprietary” in their descriptions, the primary content of commercial databases is almost entirely derived from open sources. Notwithstanding, they capture, curate, annotate, collate, integrate and index this in value-added ways (including user-friendly query front-ends and customer-specific APIs) to justify subscription costs. The second aspect is that by virtue of being able to apply more internal resources than open databases, their statistics give some indication of where the practical upper limits might lie. The third aspect is that they can give insights into the challenges of extraction, although technical details of how this is done are sparingly presented externally.

The largest relevant commercial source is CAS-SciFinder [11] While the mmoa may be indexed (i.e., providing C-P mappings) it does not include a complete DARCP capture. Consequently, this has to be classified as primarily D-C-only source. By November of 2019 SciFinder reached 157 million unique organic plus inorganic substances, having passed 100 million in June 2015. While some of these are virtual structures (i.e., never been synthesised) this large enterprise (with over 4,500 employees according to LinkedIN) has the de facto largest searchable collection of small-molecule structures extracted from papers, patents and other sources. A presentation from 2016 declared that in the first 7 months of that year ≈10.5 million substances were extracted from ≈0.5 million patents and ≈1.0 million documents. In addition, ≈75% of current novel structures are from patents. However, the 157 million is exceeded by the latest public UniChem release of just under 160 million [12]. In addition, a 2019 scaffold diversity analysis stringently filtered the CAS collection down to only ≈30 million compounds with direct links to literature and patents [13]. Since its first release in 2009 Elsevier Reaxys has emerged as another large-scale D-C capture endeavour, the statistics and search characteristics of which have recently been compared with SciFinder [14]. It has reached 31 million structures but also subsumes PubChem which brings it up to 105 million.

The two leading commercial sources that capture DARCLP at scale are the Global Online Structure Activity Relationship Database (GOSTAR) [15] from Excelra (formerly GV000Bio) and Elsevier Reaxys Medicinal Chemistry [16]. The current statistics for these are shown in Table 1.

**Table 1:** Statistics of GOSTAR (top row, from their website [15]) and RMC (from the information sheet [16]).

compounds (millions)	bioactivities (millions)	binding assays (millions)	targets (1000s)	papers (1000s)	patents (1000s)

7.8	9.7	8.7	9	191	76
6.8	35.2	–	27	370	133

The GOSTAR numbers have a more detailed breakdown in a paper from 2013 (see Table 1 in that reference) which includes the calculated averages of 12 compounds per paper and 43 per-patent [17]. GOSTAR’s compound total has doubled in the intervening six years but the extraction averages and ratio of compounds from papers: patents of ≈1:2.7 recorded in 2013 are likely to be similar. Comparable metrics for RMC curation have not been disclosed so it remains unclear what procedural differences that might explain their considerably larger activity, target and document counts compared to GOSTAR but connected to a million less compounds. Notwithstanding, using nominally the same medicinal chemistry corpus the extracted chemical structure ratios between SciFinder, Reaxys, GOSTAR and RMC are very approximately 30:30:8:7. Several technical differences may explain these ratios but the most important is the primary focus of the latter two on full DARCLP and SAR capture rather than just D-C. This selectivity in the choice of which journals and patents are curated, maintains the quality of target and activity mappings.

### Public DARCP resources

The first relevant web-instantiated curated resource, BindingDB, was published in 2001 [18]. This was followed by the IUPHAR Ion Channels Compendium of papers in 2003. This had developed into the IUPHAR-DB website by 2009 and was updated to the current IUPHAR/BPS Guide to Pharmacology (GtoPdb) by 2012 [10]. That same year also saw the first ChEMBL publication for which the website was live by 2010 [19]. All three of these resources focus on expert-curated DARCP extractions from the literature. In addition, PubChem, first appearing in 2004 has now become the de facto global hub for DARCP because all the three databases above submit their structures that are integrated with ≈700 other sources [20]. Comparative statistics of the four are shown below in Table 2.

**Table 2:** Content statistics for three DARCP sources and PubChem.

database	reference	compounds	nioactivities	targets	papers	patents

PubChem (9/19)	[21]	96 million	268 million	18000	14.2 million	3.2 mil
ChEMBL(release 25)	[22]	1.9 million	15.5 million	124000	72000	–
BindingDB (9/19)	[23]	772000	1.7 million	52000	29000	–
BindingDB patents	[24]	225000	406000	1000	–	3000
GtoPdb (2019.4)	[25]	75000	17000	20000	11000	600

**Figure 2:**
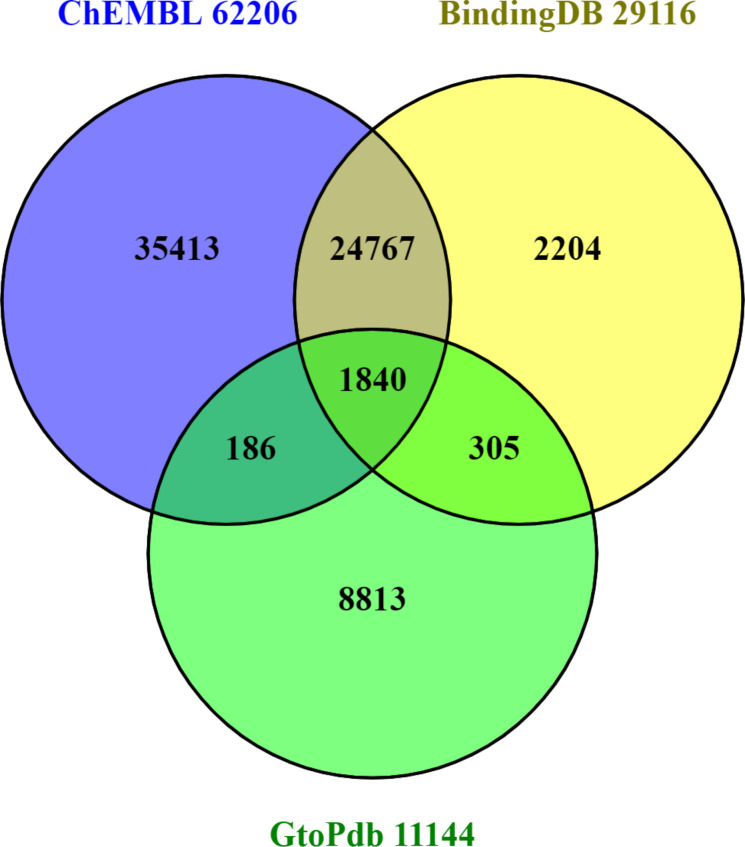
PMID content with totals appended to each segment. Those for ChEMBL were downloaded from European PubMed Central (EPMC) via the query (HAS_CHEMBL:y). For BindingDB the list was provided with courtesy of Michael Gilson. For GtoPdb the list was downloaded via the PubMed connectivity for the SIDs. The OR union is 73500 PMIDs (but all three sources also curate a proportion of DOI-only documents).

As for the commercial sources, comparing content statistics between databases is not straightforward because the numbers in Figure 2 were generated in slightly different ways. Not all the nuances can be addressed here but some salient ones can be pointed out. Moving across the columns there is an element of circularity in the compounds. The first reason is that ChEMBL subsumes 0.53 million compounds from confirmed PubChem BioAssays and 1.3 million curated from papers. The second reason is that BindingDB and ChEMBL have a reciprocal mirroring collaboration where BindingDB subsumes the protein target assay results from ChEMBL and the latter subsumes BindingDB patent extraction data (e.g., the 137,000 compounds in release 25). This is separated from their total data counts in rows three and four. It also means that the overlap of compound structures, target and document identifiers between the two sources have extensive circularity (but some are independently curated). The PubChem figures for bioactivities seem large because these are factored by substances not compounds, whereas ChEMBL (as the dominant contributor to PubChem BioAssay) collapses their assay counts to compounds. For GtoPdb the lower count reflects the curation of mainly lead compounds with curated binding constants from papers.

The 18000 targets in PubChem include automated assignments that result in an element of over-counting. Those in the other three sources are classified manually and have species-specific cross-references in UniProt [26]. These give the following human Swiss-Prot counts of 3644, 2585 and 1457 for ChEMBL, BindingDB and GtoPdb, respectively. We can see the document counts for the curated sources in column five of Table 2. From the ChEMBL release notes their literature extraction average out at ≈15 compounds-per-document (n.b., the majority will have ARCP connectivity but some have only non-bioactivity A-R data such as plasma clearance).

### Content overlaps and differences

**Figure 3:**
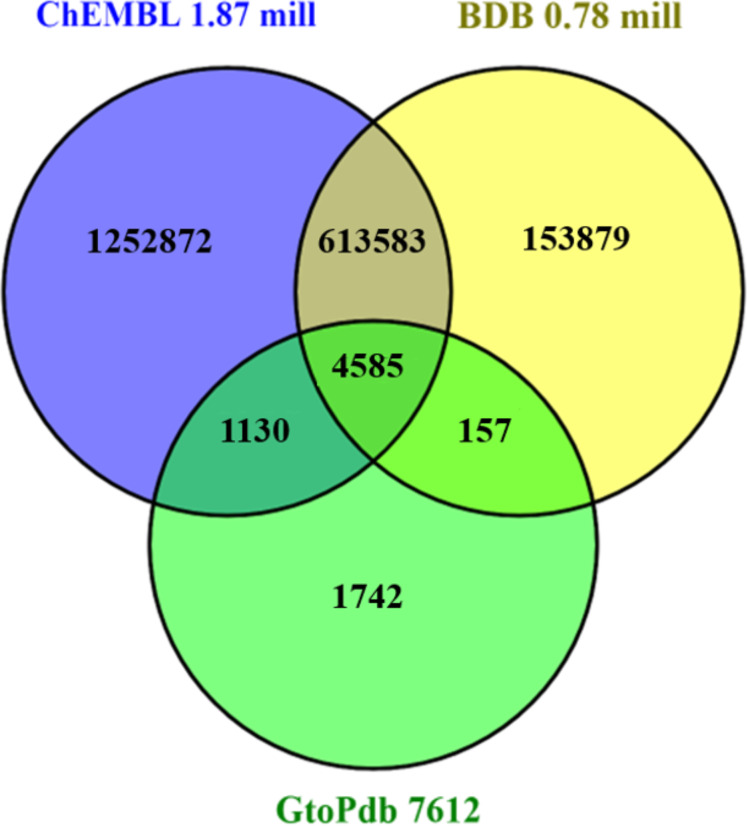
Chemistry content comparison for the three sources with totals appended to each segment, selected and downloaded as PubChem CID lists. The OR total is 2,033,127. Note that the uniqueness in the Venn sectors is just between these three. Overall uniqueness in PubChem as whole is 266000, 49000 and 143 for ChEMBL, BindingDB and GtoPdb, respectively.

**Figure 4:**
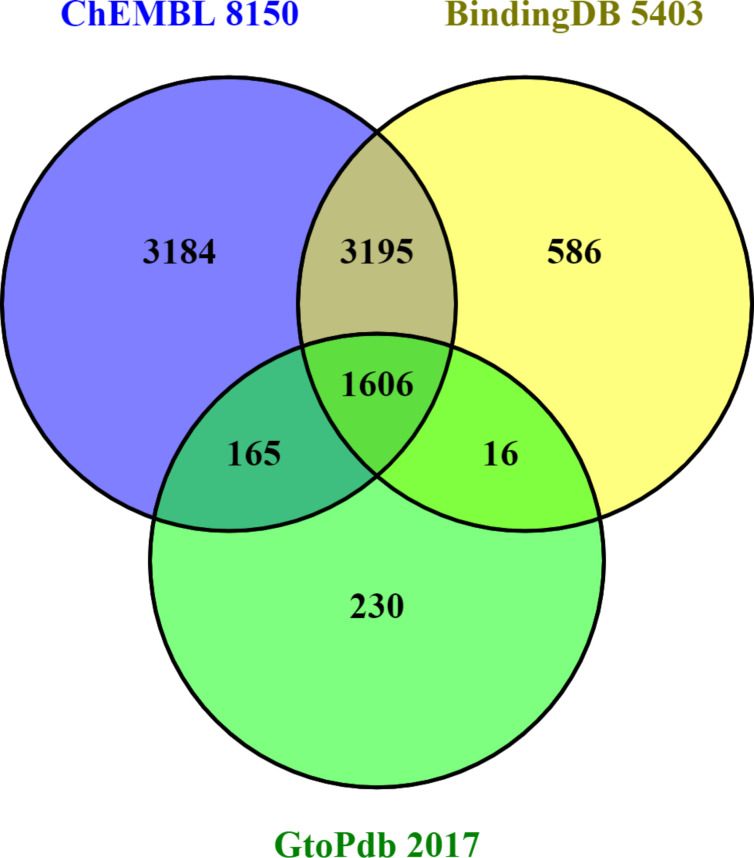
Target comparisons with totals in each segment. These were downloaded as UniProt ID lists selected via the chemistry cross-references in UniProt. These include all species but, as expected, human and rodent predominate in each case. The total of human proteins covered by the three is 3745 representing 18% of the UniProt 20,365 proteome.

Despite differences in the way their internal statistics are computed, standardised content comparison between open databases can use outputted lists for D,C and P (comparing A-R is not so straightforward). The intersects and differences between these are shown in the series of three Venn diagrams (Figures 2–4). See also Supporting Information File 1 for technical details on how these were prepared.

While there are technical caveats, we can briefly consider the implications of Figures 2–4. The PMID capture in Figure 2 shows a pattern of intersects and differences that is to some extent reflected for the other entities also. Each indicates some unique capture but ChEMBL and BindingDB overlap for ≈25000 papers. Despite being the smallest of the three, GtoPdb shows proportionally more unique PMIDs. This is substantially due to the curators adding additional references into the SID records beyond those from which the binding data were extracted (e.g., in vivo and clinical reports published after the initial in vitro results). Notably, the public total from all four of ≈75000 is less than 50% of the journal document counts declared by the two commercial sources (Table 1). While the limited resources of the public sector are clearly a factor, it would be informative to know explicitly what was behind the differences. Journal selectivity is likely to be dominant but other factors may come into play.

The chemistry content in Figure 3 shows similar disproportionation with ChEMBL, as expected, dominating unique content at over 1.2 million. While this is skewed by the BioAssay subsumation of ≈0.5 million, most will be a consequence of extracting ≈35000 unique PMIDs. For BindingDB most of their 153000 unique structures are from the ≈200000 protein-ligand binding data points that were curated from 1,100 US Patents during 2019 (n.b., these will eventually be subsumed into ChEMBL release 26). We can further rationalise the proportionality between compounds and PMIDs by noting that GtoPdb extract on average ≈1 lead compound per paper, ChEMBL ≈14 per paper with BindingDB extracting similar numbers from papers but ≈40 per patent.

The differences in target coverage (i.e., as “P” in DARCP) shown in Figure 4 are noteworthy and persist despite the ChEMBL/BindingDB selective mirroring. As for PMID coverage it would be useful to know which types of selectivity were responsible for this divergence in connectivity. For BindingDB some unique proteins are likely to be patent-only but exploring further causes of complementary target coverage are outside the scope of this work.

### Journals connecting to PubChem

As anomalous as it may seem, no individual journals have put in place a direct feed of author-specified DARCP into PubChem BioAssay (or any other database for that matter). Historically, four journals have initiated D-C feeds in PubChem but two of these, *Prous Science Drugs of the Future* and *Nature Communications*, ceased in 2012 and 2014 respectively. This has left only *Nature Chemical Biology* and *Nature Chemistry* as still active with 12481 and 15276 author-specified CID structures respectively (plus some on-hold submissions) but the latter journal does not typically include bioactivity reports. Some Elsevier journals do list CIDs in their abstracts but without submitted links.

**Figure 5:**
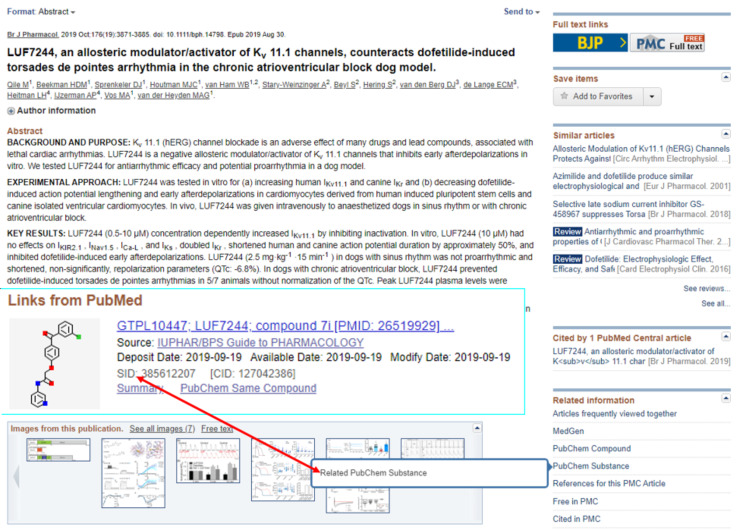
Connectivity for PMID 31339551. The lead structures from the paper, LUF7224 was curated by GtoPdb as ligand ID 10447 for which the DARCP is thus annotated (with P being Kv11.1 in this case). The submission of PubChem SID 385612207 by GtoPdb is indexed in Entrez with a live PubChem link from the PubMed right hand facet. The arrow indicates the reciprocity in that users can navigate “in” to the system via GtoPdb or “out” either via the CID or the PMID.

One journal that has pioneered a first approximation to DARCP flow into PubChem is the *British Journal of Pharmacology* (although the links are technically indirect) [27]. The annotation task was initially done by editors but since 2016 authors have been incorporating GtoPdb ligand and target identifiers in their text that became clickable out-links in the published HTML and PDF versions. This has the additional advantage of setting up a virtuous circle of reciprocal connectivity with PubMed where DARCP curated by GtoPdb has been submitted to PubChem. This is outlined in Figure 5.

### Anomalies in the system

The wider informatics ecosystem exhibits a range of quirks related to DARCP and DC capture. These can complicate connectivity, confound standardisation and make navigation difficult, especially where they are non-obvious. The technical decisions that have caused such anomalies were generally been made to accommodate different submitter requirements (i.e., no one is trying to make the system more complicated, it just seems that way). The following is a selection:

1.) PubChem presents users with the complexity of parallel systems of D-C connectivity [28]. For medical subject headings (MeSH) the publication links are biased towards common name matches in many papers (e.g., the MeSH category for chemicals and drugs links 127000 PubChem CIDs to over 14 million PMIDs). Somewhat surprisingly, the largest D-C source by far is the IBM automated patent extraction system. This has operated on not only patents but also PubMed (plus MeSH terms in those abstracts) as well as full text from PMC articles. By 2016 this was responsible for 56% of all PMID-CID mappings (although IBM made what may have been their final submission in 2017). PubChem has a third substantial category of D-C connectivity from the publishers Springer, Thieme and most recently Wiley. These three sources have added document links for 660, 740 and 118 thousand CIDs respectively (with an overlap of only 74000). However, those having DOI-only document links are not connected into Entrez. They are made accessible via cross-references in the CIDs for Thieme and Wiley but only via SIDs for Springer. Since these publishers have provided a proof of concept for these D-C efforts it is to be hoped that they will be followed by equivalent undertakings from other chemistry publishers, for example ACS, Elsevier and ChemRxiv.

2.) The three DARCP sources with conceptually equivalent curated links (Table 2) are indexed within the NCBI systems in different ways. GtoPdb links into Entrez via reciprocal connectivity between 11000 PMIDs and 9800 SIDs. It also has just under 2000 BioAssay links by target class. ChEMBL has just over 1 million BioAssay entries largely as per publication indexing and also has its own target hierarchy in PubChem. While the SIDs have no PMID links 34000 of the ChEMBL-extracted PMIDs are indexed in Entrez but only via BioAssay. BindingDB has neither extensive BioAssay links nor Entrez SID connectivity. However, the 28000 curated PMIDs in this case are connected into the NCBI system as a LinkOut source.

3.) As a commendable initiative the *Journal of Medicinal Chemistry* requires authors to provide SMILES [29] and in some cases, they may also add activity values, as supplementary data. These are made available as comma-separated (.csv) files. However, while this was envisaged to facilitate automated extraction, no one actually does this (or at least has not openly surfaced the results). These files thus useful contain C-R but A and P remain in the paper (although DARCP from this journal is extensively curated by GtoPdb, ChEMBL and BindingDB).

4.) Despite the pioneering efforts of *Nature Chemical Biology* there are caveats associated with their D-C mappings. The first is that in their SID records they index DOIs in the Depositor Comments but not PMIDs. This means there is no connection into the Entrez system (although this may have been an expedient choice to avoid the lag time associated with post-publication PMID assignments). As another quirk, there are 2,447 structures submitted by the journal that do not have an exact match to those extracted by the Springer automated pipeline for the same documents. It would be advantageous (including increasing traffic to the journal) if they could extend the author data submissions to enable full DARCP representation in PubChem BioAssay for suitable data sets.

5.) The transfer of data from the literature into on-line open resources (by an individual or a curation enterprise) could conceivably come up against copyright issues. The complexities of permissions to mine scholarly content were reviewed in 2016 [30]. It was reported therein that small amounts of data (e.g., presumably encompassing DARCP) would not generally be considered “creative expression” and thus should be exempt from copyright. We can also note that, after the fact, many public databases have been adding both curated and automatically mined “data facts” for well over a decade.

6.) The extraction of DARCP and D-C connectivity from patents presents a number of anomalies specific to this document type. The first is that, compared to journal articles in which a proportions of the same data are later republished, in terms of compound structures in-common the appropriate PubChem query records a CID intersect of 29% between ChEMBL and all the major automated patent chemistry extraction sources adding up to 29.7 million), the document corpus has no paywalls. This means it is not only free-to-mine but also the HTML (before hamburgerisation) available from the USPTO greatly facilitates automated extraction. The second is that, in contrast to the commercial DARCP curation efforts by Excelra and Elsevier (implying the perceived high value), public extraction of patent ARCP is limited almost exclusively to BindingDB. However, as a consequence of being free-to-mine a number of operations have carried out public large-scale automated D-C extractions. These include, SureChEMBL [31], IBM, World Intellectual Property Organization (WIPO) and most recently Google Patents. However, the problem arises in PubChem and other sources) of what could be called “swamping” from the continual re-indexing (i.e., D-C linking) of common chemistry and structures without any ARCP data. As an example, in PubChem CID2244 for aspirin there are 143,180 connections to patent documents.

## Conclusion

Comparing the historical connectivity between bioinformatics and cheminformatics points towards the root of the problems we currently face. Over more than three decades the links between sequence data and the literature have become a blazing success, first for molecular biology followed by genomics. This was driven mainly by the combination of journal mandates for author inclusion of sequence accession numbers and somewhat later, pointers to genomic and expression data sets. This has needed herculean technical integration efforts not only from the NCBI Entrez system and the equivalent EBI resources but also global coordination by the International Nucleotide Sequence Database Collaboration (INSDC) [32]. While compliance is not 100%, extensive literature and data set connections are now captured by both PubMed, PMC and EPMC.

The paradox is that no open equivalent ever emerged in the chemistry domain, in part due to the dominance of SciFinder. Thus, despite PubChem CIDs appearing in 2004 and the InChI identifier being implemented in 2013, publishers (with a few exceptions) have neither mandated nor encouraged the inclusion of open, machine readable chemical representations and/or open chemical database identifiers in their journals (external to the PDFs). The consequent shortfall for chemistry capture in general and DARCP in particular, shows no signs of diminishing. A rough estimate, derived from comparing commercial numbers from Figure 3 with public ones in Table 1, would be a chemical structure ratio of roughly 2 million to 7 million (i.e., a public shortfall in the order of ≈5 million although the major part of the later comes from patents).

An important corollary is that despite progress in automated chemical and biomedical entity recognition from text (e.g., via Natural Language Processing, NLP) [33] the fully automated extraction of explicit DARCLP relationships from documents has not yet been achieved (although AI efforts are doubtless pushing towards this). This stands in contrast to the ability of biocurators to discern such relationships from a paper in minutes (but needing extra minutes for a patent) [34]. The expansion of automated D-C capture in PubChem (e.g., by Springer, Thieme and Wiley) as well as automated chemical look-up in PMC and EPMC are certainly welcome developments. Notwithstanding, the associated ABNE problems severely limit knowledge distillation from D-C connections alone.

So, where do we go from here? The good news is that GtoPdb, ChEMBL and BindingDB should continue their expert capture role. The bad news is that it looks like, even by 2020, no journal will yet have instigated a formal process to extract DARCP and pipe it directly into open databases. One can only surmise that there is neither sufficient publisher “pull” nor author-incentivised “push” to make this happen. An alternative solution would be for authors to independently facilitate the transfer of their own annotated DARCP data into, for example, PubChem BioAssay. While the key connectivity to a PMID (via Entrez) could be added later, the necessary database submissions (possibly directly from an ELN) could, in principle, be de-coupled from the publishing process and thereby bypass PDF-entombment. Here again, we come up against the impasse of which stakeholders would value this high enough to instigate it.

Notwithstanding, we can take a more optimistic look at recent developments in the wider context of knowledge sharing including semantics and linked data that have the potential to improve the situation for DARCP capture. These include open data [35], open access (OA) [36], FAIR (findable, accessible, interoperable and reusable) [37–38], resource description framework (RDF) [39] and [40] WikiData [40]. While there is certainly momentum behind these trends, the persistence of publisher paywalls still remains a serious obstacle (e.g., of the 62000 papers curated by ChEMBL in EPMC only 85000 are full-text and only 600 OA). Strong community adoption (including from publishers) is also being seen for FAIR, which, in principle, should encompass accessibility to D, A, R, C and P (even if not their explicit connectivity) Planning is underway for the capture of FAIR data in various repositories (e.g., Figshare) but quite how this would practically expedite the flow of connected DARCP into major databases (including core resources of the ELIXIR - distributed infrastructure for biological data [41]) is not yet clear. Another new development in the list, WikiData [40], is a community-maintained knowledge base that builds on the principles of FAIR. Here again, we will have to see how the practicalities of crowdsourcing DARCP curation and feeds into open databases can be accomplished.

## Supporting Information

File 1 Technical details.
