# Supplementary material for: Opening up connectivity between documents, structures and bioactivity
Source: Beilstein J Org Chem. 2020 Apr 2;16:596–606. doi: 10.3762/bjoc.16.54 (PMC7136548; doi:10.3762/bjoc.16.54)
Supplement: File 1 — Technical details. [file Beilstein_J_Org_Chem-16-596-s001.pdf]

BEILSTEIN JOURNAL OF ORGANIC CHEMISTRY

Supporting Information

for

Opening up connectivity between documents, structures and bioactivity

Christopher Southan

Beilstein J. Org. Chem. **2020,** *16,* 596–606. doi:10.3762/bjoc.16.54

Technical details

License and Terms: This is a supporting information file under the terms of the Creative Commons Attribution License (http://creativecommons.org/licenses/by/4.0). Please note that the reuse, redistribution and reproduction in particular requires that the authors and source are credited.
The license is subject to the *Beilstein Journal of Organic Chemistry* terms and conditions: (https://www.beilstein-journals.org/bjoc)

This document describes how to reproduce (or update and extend as desired) the intersect data displayed as Venn diagrams for the three sources and three key entity types compared in Figures 2, 3 and 4 in the paper.

Intersecting PubMed IDs (Fig.2)

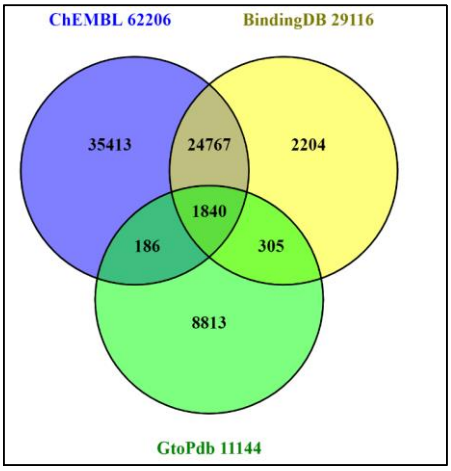

The objective here was to compare the journal publication identifiers that each source used for curatorial extraction of DARCP. This was standardised to PubMed IDs (PMIDs) but it should be noted that all three sources also extract a proportion of DOI-only papers that are not counted in this analysis.

ChEMBL: Since the PMIDs are only indexed in PubMed BioAssay but not NCBI Entrez the European PubMed Central (EPMC) indexing has to be used. The query (HAS_CHEMBL:y) returns 64580 results (Feb 2020) but because of the 50000-record limit this has to be downloaded in two parts (easily partitioned by date). The difference between this and the 69914 publication records in ChEMBL 25 is assumed to be the DOI-only papers.

BindingDB: in this case a current list of 29116 PMIDs was supplied by Prof. Michael K Gilson. However, it is also possible to get a slightly smaller set (probably an update lag) via the NCBI Linkout system. The query https://www.ncbi.nlm.nih.gov/pubmed/?term=loprovBindingDB[SB] returns 27796 PMIDs (Feb 2020)

Guide to Pharmacology (GtoPdb): This is the only one of these three to be directly indexed in NCBI Entrez. However, accessing a PMID listing need two queries. The first is a source select in the PubChem Substance database (i.e. the SIDs). This needs to be followed by linking to PubMed. The interface format for these two queries are shown below.

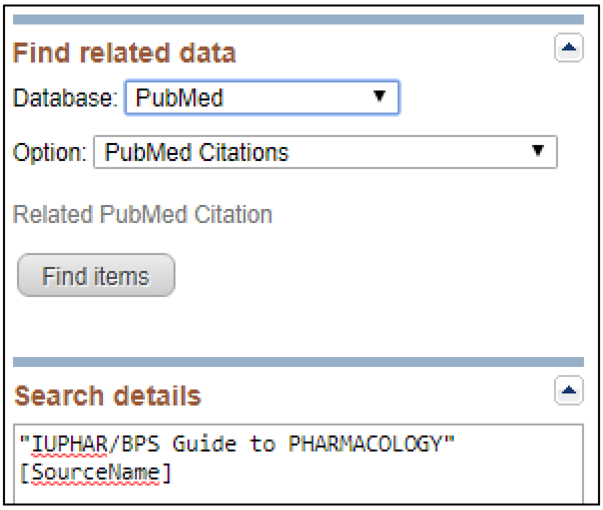

The PMIDs can then be download via the settings below

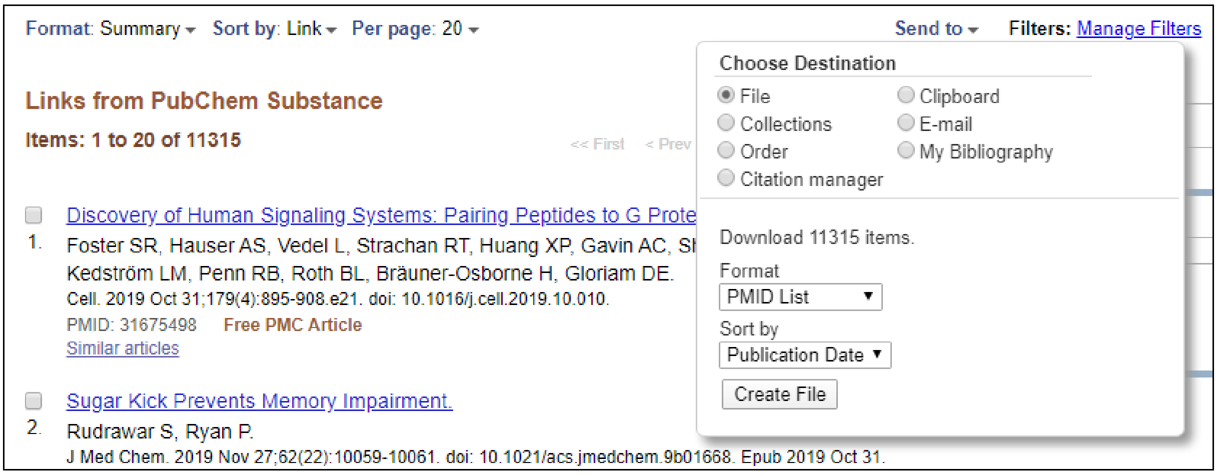

This yields 11315 PMIDs (synched with the GoPdb release 2019.5 of Nov 2019) but there are two caveats. Firstly, this is maximal list in that it includes secondary references such as review articles and clinical reports referenced against the GtoPdb ligands in addition to the smaller set of primary papers from which the quantitative interaction data were extracted. Secondly, this includes those substances (as SIDs) that will not form chemical structures (e.g. antibodies, small protein ligands and large peptides). It is possible to access only those papers from which quantitative interactions were extracted via the EPMC “External Links” query (LABS_PUBS:"1969") that lists 6753 PMIDs.

The Venn diagram can be made with the Venny 2.1 tool interface https://bioinfogp.cnb.csic.es/tools/venny/. For Figure 2. the inputs are shown below.

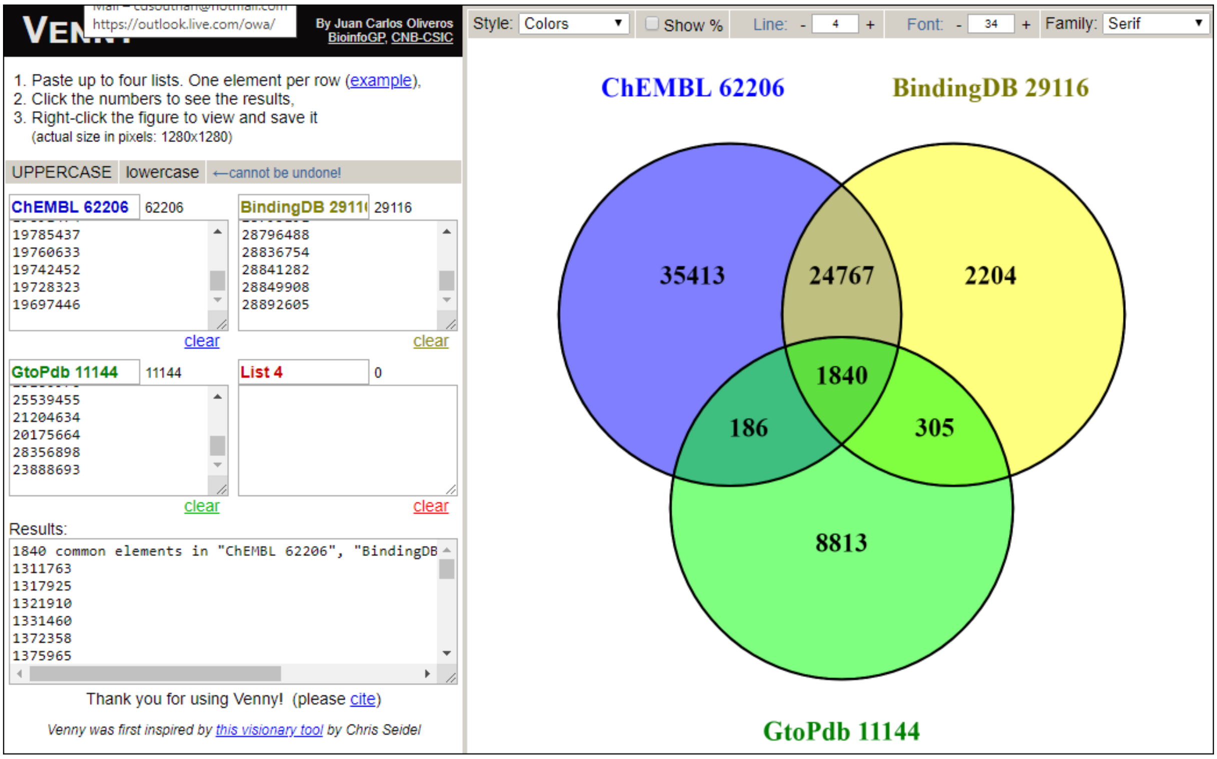

Note that Venny (also used for Figure 4) has a number of useful features for this kind of analysis. Firstly, any standardised string lists can be used. Secondly, multiple lists pasted in the same box are de-duplicated. Thirdly output lists for each segment can be generated for inspection, further analysis (e.g. by uploading to PubMed) and the interpretation of inter-database differences. For example, the snapshot above lists the "Results" of the 1840 PMIDs in-common between all three sources (but not necessarily with identical extractions).

Intersecting PubChem CIDs (Figure 3)

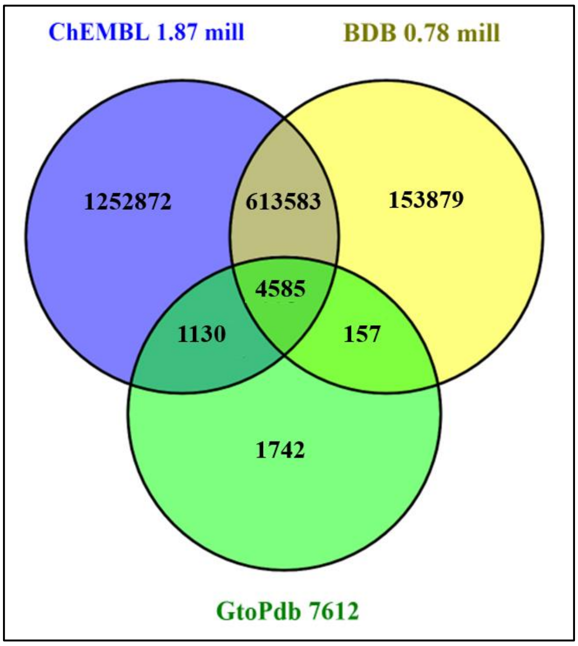

The practical issue here is that the numbers exceed the capacity of Venny. However, because PubChem indexes the CIDs in a standardised way for three the sources, Venn-type intersects can be generated via the PubChem compound interface https://www.ncbi.nlm.nih.gov/pccompound . This has the key feature of being able to execute Boolean operations on query history. Venns can thus be generated but arranging and bracketing the correct AND and NOT becomes challenging for more than three sources. Example results that can be extended for the completion of Figure 3 are shown below.

Query	Items found
Search ("IUPHAR/BPS Guide to PHARMACOLOGY "[SourceName]) NOT ("ChEMBL "[SourceName]) NOT ("Bindingdb "[SourceName])	1779
Search ("Bindingdb "[SourceName]) AND ("IUPHAR/BPS Guide to PHARMACOLOGY "[SourceName]) NOT ("ChEMBL "[SourceName])	161
Search (("Bindingdb "[SourceName]) AND "ChEMBL "[SourceName]) AND "IUPHAR/BPS Guide to PHARMACOLOGY "[SourceName]	4652
Search "Bindingdb "[SourceName]	804851
Search "ChEMBL "[SourceName]	1872170
Search "IUPHAR/BPS Guide to PHARMACOLOGY "[SourceName]	7674

A useful tip is to use the “Advanced Search Builder” to execute the queries via the “Add to history” (i.e. retuning just the count as opposed to the full Entrez rendering) rather than using the “Search” button that may time out. Note that, a) consequent to PubChem updates for BindingDB and GtoPdb the numbers have changed slightly between October and February b) for consistency of presentation the PubMed data has been pasted-up in Figure 3 to mimic the Venny output style.

Intersecting protein IDs (Figure 4)

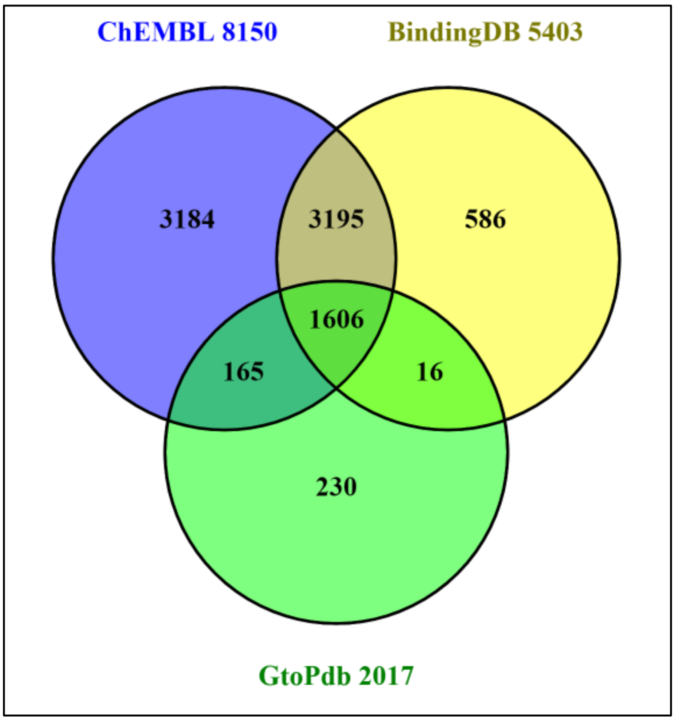

The generation of this figure was enabled by UniProt and Venny. Along with other chemistry cross-refence sources, the three databases are shown in the menu snapshot below.

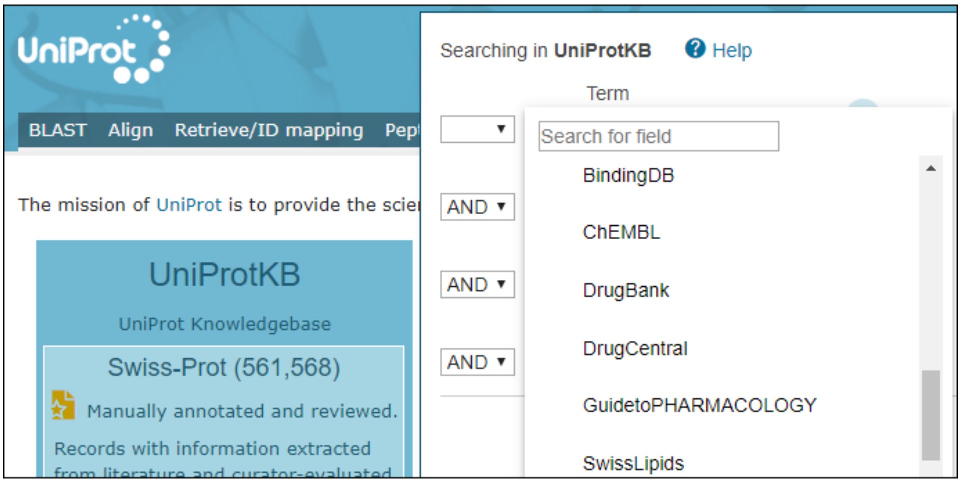

The complete select string for Swiss-Prot entries in GtoPdb is shown below.

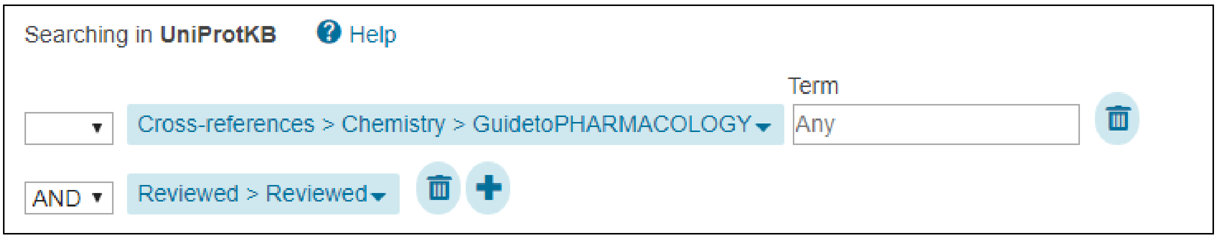

And the download options can be configured as below

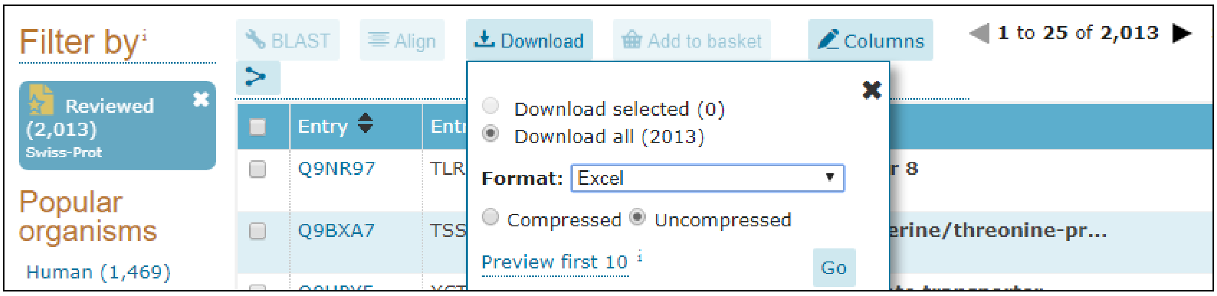

Downloading all three lists and inputting the UniProt IDs into Venny (analogously as for Figure 2) produced Figure 4. Beyond simple reproduction the analysis can be extended in many ways. For example a) species can filtered to just human targets b) results from any segment of the Venn can be uploaded to UniProt for inspection and filtration for any combination of properties or cross-references, c) Venny can take four list inputs so the analysis can be extended to adding other chemistry-mapping databases such as DrugBank or DrugCentral.
